# Complete chloroplast genome of the hot desert herb *Fagonia indica* (Zygophyllaceae) from south-central Arabia

**DOI:** 10.1080/23802359.2019.1687358

**Published:** 2019-11-08

**Authors:** Mohammad Ajmal Ali, Mohammad S. Elshikh, Soo-Yong Kim, Fahad Al-Hemaid, Joongku Lee, Dorjay Lama, Abhijit Chhetri, Tapan Kumar Pan

**Affiliations:** a Department of Botany and Microbiology, College of Science, King Saud University, Riyadh, Saudi Arabia;; b International Biological Material Research Center, Korea Research Institute of Bioscience and Biotechnology, Daejeon, Republic of Korea;; c Department of Environment and Forest Resources, Chungnam National University, Daejeon, Republic of Korea;; d Department of Microbiology, St Joseph’s College, Darjeeling, India;; e University Department of Botany, Tilka Manji Bhagalpur University, Bhagalpur, India

**Keywords:** Chloroplast genome, *Fagonia indica*, Zygophyllaceae, Krameriaceae, South-Central Arabia

## Abstract

The complete chloroplast genome sequences of the hot desert herb *Fagonia indica* (Zygophyllaceae) is being reported in this study. The total plastome length was 128,379 bp (GC content 34.02%). The gene order in *F. indica* was found similar to the angiosperm except for the loss of one copy of the IR and by the presence of a single, large inversion that reverses the order of the genes between *rbcL* and *rps16.* A total number of 115 unique coding genes which includes 80 protein-coding gene, 31 tRNA genes, and 4 rRNA genes were annotated. The phylogenetic analysis of representative plastomes from the Roisds revealed two distinct clades of Krameriaceae and Zygophyllaceae.

The genus *Fagonia* L. (family Zygophyllaceae), consist of c. 35 species are herbs or shrubs of about 60–100 cm maximum in length and width, respectively, possess disjunct distribution (Beier 2005). *Fagonia indica* Burm.f. is a thorny wild medicinal herb (Qureshi et al. 2016) growing widely in Asian and African deserts (Beier 2005) including the Empty Quarter (Mandavil 1986) where either no rain for several years or less than 35 mm. The phylogenetic relationships of the two sister families, e.g. Zygophyllaceae and Krameriaceae (Tao et al. 2018) under the order Zygophyllales have often been controversial (Granot and Grafi 2014). Hence, we herein for the first time sequenced the complete plastome of *F. indica*, and assessed the systematic relationships of the order Zygophyllales.

The total genomic DNA of *F. indica* was isolated from fresh leaves [Voucher: MAA 14 (KSUH)] collected from Riyadh, Saudi Arabia (24°43′15″N 46°37′5′E″) using DNeasy (#QIAGEN) kit, and were sequenced at Illumina sequencing platform. A total number of 51,955,132 paired-end reads of 151 bp were obtained. A total of 115 unique coding genes including 80 protein-coding genes, 31 tRNA genes, and 4 rRNA genes were annotated. The total plastome length (128,379 bp; GC content 34.02%, GenBank MN521457) was found consistent with the *Larrea tridentata* (DC.) Coville (family Zygophyllaceae) plastome. The gene order was found similar to angiosperm (Raubeson et al. 2007) except for the loss of one copy of the IR and by the presence of a single, large inversion that reverses the order of the genes between *rbcL* and *rps16.* The coding genes in the genome represent 73,171 bp nucleotides coding for 42,793 codons. The ML analysis of cp genes performed using MEGA X (Kumar et al. 2018) revealed two distinct clades of Krameriaceae and Zygophyllaceae, which is congruent with wood anatomy (Carlquist 2005) (Figure 1).

**Figure 1.**
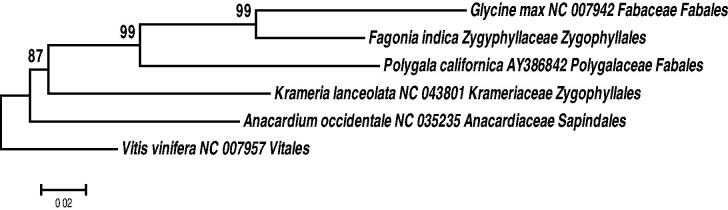
The MLT of Rosids based cp genome (the bootstrap supports in 1000 bootstrap replicates are shown above the nodes).
